# Post-Thromboembolectomy Pseudoaneurysms Affecting Below-the-Knee Arteries and Their Management Strategies: A Literature Review

**DOI:** 10.3390/jcm14072176

**Published:** 2025-03-22

**Authors:** Ákos Bérczi, Dóra Papp, Fanni Éva Szablics, Edit Dósa

**Affiliations:** Heart and Vascular Center, Semmelweis University, 1122 Budapest, Hungary

**Keywords:** thromboembolism, thromboembolectomy, Fogarty balloon catheter, pseudoaneurysm, below-the-knee arteries, endovascular intervention, surgical repair

## Abstract

Pseudoaneurysms resulting from Fogarty balloon catheterization for thromboembolism (termed post-thromboembolectomy pseudoaneurysms [PTPAs]) are rare but pose significant clinical challenges, particularly when they affect below-the-knee arteries. The underlying pathophysiology of PTPAs involves arterial wall injury, leading to blood extravasation and the formation of a pseudoaneurysm sac. The presentation of PTPAs varies but is often characterized by localized pain, swelling, and a palpable pulsatile mass, which may appear immediately or be delayed. Diagnostic modalities such as duplex ultrasound, computed tomography angiography, and digital subtraction angiography are essential for accurate detection and characterization. Management strategies for PTPAs range from conservative observation to radiological interventions and open surgical reconstruction. The choice of treatment depends on factors such as the size and anatomical location of the pseudoaneurysm, as well as the patient’s overall health status. This review synthesizes case reports and highlights the critical importance of prompt diagnosis and individualized treatment strategies. Additionally, it underscores the need for ongoing research, particularly in complex cases requiring a combination of approaches, to refine treatment protocols and improve patient outcomes.

## 1. Introduction

Acute limb ischemia is defined as a sudden or rapidly developing reduction in limb perfusion, typically leading to the onset or worsening of symptoms and often posing a threat to the viability of the affected extremity [1,2]. The etiology of acute limb ischemia can be categorized into three main causes: thrombosis, embolism, and trauma. Of these, the first two account for the majority of cases. The primary mechanism underlying acute limb ischemia is arterial occlusion, which may involve the blockage of a native artery, a vascular bypass graft, or a stent/stent graft. Although rare, extensive venous occlusion can also result in extremity ischemia (phlegmasia). Acute limb ischemia is associated with high morbidity and mortality rates, with arterial occlusion leading to acute lower extremity ischemia occurring in approximately 1.5 cases per 10,000 persons per year [3].

The management of acute limb ischemia due to embolism is centered on rapid re-vascularization of the affected extremity to reduce morbidity and prevent limb loss. Standard initial medical treatment includes anticoagulation with intravenous heparin to prevent thrombus expansion [1]. For patients with suitable anatomy and minimal comorbid risks, endovascular therapies—such as catheter-directed thrombolysis and percutaneous mechanical thrombectomy—are often effective in restoring blood flow [4]. However, when limb viability is immediately threatened or when large emboli are located near major arterial branches, open embolectomy is preferred for its proven efficacy in rapidly reestablishing circulation [1]. The choice between endovascular and surgical intervention depends on several factors, including the duration of ischemia, the location of the embolic obstruction, and the patient’s overall stability [1].

Surgical embolectomy, particularly using a Fogarty balloon catheter, has long been a standard procedure for treating acute lower extremity ischemia caused by thrombosis and embolism [5,6,7,8]. While effective, this procedure carries risks such as the development of pseudoaneurysms [9,10,11,12]. Post-thromboembolectomy pseudoaneurysms (PTPAs) most commonly occur in the infrapopliteal arteries, including the anterior tibial, posterior tibial, and peroneal arteries [9,10,12,13,14,15]. The etiology of these PTPAs is believed to stem from arterial wall injury, likely caused by the mechanical forces exerted by the Fogarty embolectomy balloon. The underlying pathophysiological process involves the disruption of the arterial wall’s integrity, leading to blood extravasation into surrounding tissue and the subsequent formation of a fibrous sac, a hallmark of pseudoaneurysm development [16].

Given the fragility of below-the-knee (BTK) arteries and their critical role in maintaining distal limb perfusion, effective management of pseudoaneurysms is essential to prevent severe complications, including limb amputation. This review evaluates the current standard of care for treating PTPAs, a known complication of Fogarty catheter thromboembolectomy in BTK arteries.

## 2. Prevention of PTPAs

To prevent the development of PTPAs, special attention to technical details is required during embolectomy. The size of the Fogarty catheter should be determined based on the size of the target vessel, and catheter changes are important when the surgeon is traversing arteries of different sizes. The amount of fluid delivered to the balloon can be maximized depending on the size of the catheter (this is indicated on the hub of each catheter). During embolus/thrombus removal, careful adjustment of balloon pressure and volume is essential, using a hand syringe to ensure accuracy. In addition, the size and position of the balloon can be verified during the procedure using a C-arm.

## 3. Symptoms

The clinical presentation of a PTPA is characterized by localized pain, swelling, and/or a palpable pulsatile mass [10,11]. These symptoms may appear acutely, shortly after the procedure, or in a delayed manner, sometimes weeks later [9,15,17]. A delayed presentation is often attributed to a gradual expansion of the pseudoaneurysm or the development of complications such as arteriovenous fistulas or arterial rupture [9,15,17]. Symptoms may also be accompanied by signs of ischemia, including reduced distal pulses and sensory deficits, warranting further evaluation.

## 4. Diagnosis

The diagnosis of PTPA in BTK arteries requires a comprehensive approach that integrates both clinical assessment and imaging techniques. Commonly used imaging modalities include duplex ultrasound, computed tomography angiography (CTA), and digital subtraction angiography (DSA) [1,2,18,19].

Duplex ultrasound is a crucial noninvasive diagnostic tool for detecting post-thromboembolectomy complications. It provides real-time imaging and enables the identification of characteristic blood flow patterns and hemodynamic alterations. In color mode, a “yin-yang” sign is visible within the pseudoaneurysm sac, while Doppler mode reveals a “to-and-fro” flow pattern in the pseudoaneurysm neck [19,20]. This modality is particularly useful for assessing the size and morphology of the pseudoaneurysm [15]. Additionally, its accessibility and ability to guide therapeutic interventions, such as thrombin injection, support timely and effective treatment [9,11]. Ultimately, ultrasound plays a vital role in facilitating interventions, monitoring for further complications, and providing post-treatment follow-up.

When ultrasound findings are inconclusive or a detailed vascular map is required, CTA is performed [12,14]. This imaging technique provides high-resolution images that aid in assessing anatomical relationships and the extent of the lesion. A pseudoaneurysm appears as an outpouching sac with a round, smooth margin in continuity with the arterial lumen.

**Figure 1:**
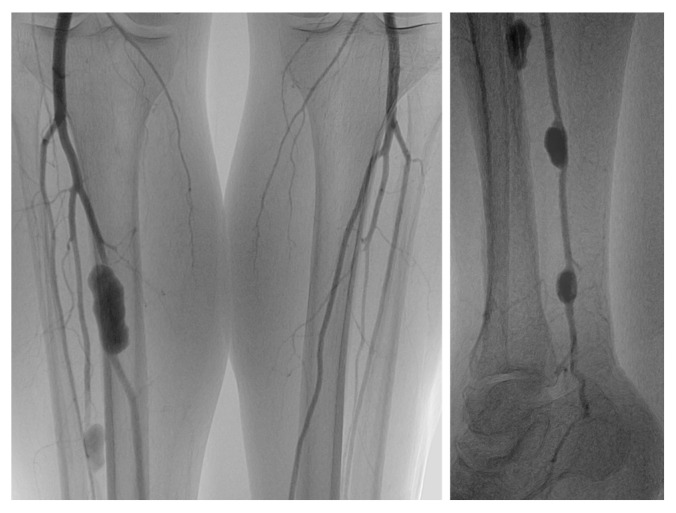
DSA images depict multiple PTPAs in BTK arteries of a 44-year-old man. These images were obtained by Edit Dósa (Heart and Vascular Center, Semmelweis University).

DSA remains the gold standard for the definitive diagnosis and treatment planning of PTPA, despite its invasive nature. It enables precise localization and characterization of the pseudoaneurysm while also providing visualization of the lesion’s flow dynamics [9,13,14,17] (Figure 1). In the surgical management of PTPAs, intraoperative angiography is valuable for confirming the diagnosis and assessing procedural efficacy.

This multi-modality approach is essential to the diagnostic process because it helps determine the most appropriate treatment strategy—whether endovascular or surgical—based on the specific characteristics of the pseudoaneurysm [20,21,22].

## 5. Treatment

The management of pseudoaneurysms following surgical thromboembolectomy varies significantly, ranging from conservative surveillance to radiological interventions and open surgical repair. Treatment selection is influenced by several factors, including the size and location of the pseudoaneurysm, the presence of associated symptoms, and the patient’s overall health status.

### 5.1. Conservative Treatment

In cases where the pseudoaneurysm is small and asymptomatic, a conservative approach with close monitoring is appropriate. This strategy includes periodic imaging to track the pseudoaneurysm’s size and detect potential complications, such as rupture or significant growth.

### 5.2. Endovascular Procedures

Endovascular or transcatheter interventions have become important, less invasive alternatives to open surgery. Coil embolization and the use of covered stents are two common techniques for treating pseudoaneurysms. These approaches offer potential advantages, including shorter procedural times, reduced post-procedural hospital stays, and faster recovery compared with traditional surgery. Coil embolization involves inserting platinum coils into the pseudoaneurysm sac or feeding artery to induce thrombosis and exclude the pseudoaneurysm from circulation. This technique is particularly useful when preserving the parent artery is not critical or feasible [17]. Alternatively, covered stents can be used to exclude the pseudoaneurysm while maintaining arterial patency, making this approach especially beneficial when parent artery preservation is essential [13].

### 5.3. Percutaneous Interventions

Percutaneous or direct puncture embolization is a less invasive alternative for treating pseudoaneurysms. This technique involves directly puncturing the pseudoaneurysm sac and deploying embolic materials, such as coils, or injecting thrombin to halt blood flow [9,10,11,12,14,17]. The thrombin injection can be performed with or without balloon occlusion of the affected arterial segment, depending on the clinical scenario and the treating physician’s preference. Balloon occlusion is useful in some cases to prevent distal embolization and to provide better control of the procedure [9,10,11,12,14,17].

### 5.4. Open Surgical Reconstruction

Open surgical intervention is often necessary for symptomatic pseudoaneurysms or those at high risk of complications. Conventional surgical techniques include direct repair of the arterial defect, pseudoaneurysm resection, and bypass or interposition grafting to restore distal perfusion [15]. However, surgical treatment presents challenges, such as the technical difficulty of accessing small-caliber BTK arteries and the risk of postoperative complications, including infection, graft failure, and re-occlusion. Despite these challenges, open surgical reconstruction remains the preferred treatment option, particularly when less invasive methods are not feasible or have failed.

### 5.5. Hybrid Techniques

In complex cases, a combination of endovascular and open surgical approaches may be required. Hybrid techniques allow for the immediate endovascular management of the pseudoaneurysm, followed by definitive surgical repair if necessary. This approach is particularly beneficial for critically ill patients because it minimizes the procedural time and physiological stress [13].

## 6. Complications and Prognosis

The treatment of PTPAs carries inherent risks, including infection, distal embolization, pseudoaneurysm recurrence, and repair failure. Additionally, the risks associated with endovascular and surgical procedures—such as contrast-induced nephropathy, access site complications, and anesthesia-related issues—must be considered. The prognosis largely depends on the timeliness of diagnosis and intervention, as well as the patient’s overall health status. Early recognition and management are crucial to preventing severe outcomes, including limb loss. Follow-up imaging is essential to ensure long-term treatment success and to monitor for recurrence or other complications [13].

## 7. Review of the Literature

**Figure 2:**
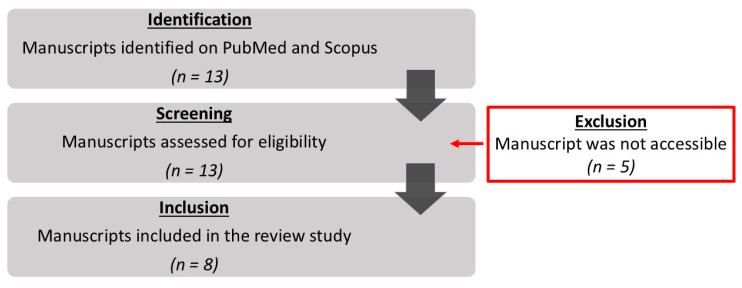
PRISMA flow chart of study selection.

A non-systematic search of the PubMed and Scopus databases was conducted to identify relevant studies published from January 2000 to May 2024. The search strategy encompassed a combination of Medical Subject Headings (MeSH) terms and text words related to PTPAs affecting BTK arteries and their treatment modalities. The following MeSH terms were utilized: “pseudoaneurysm”, “false aneurysm”, “below-the-knee”, “crural”, “peroneal”, “post-embolectomy”, “post-thromboembolectomy”, and “Fogarty”. The literature selection was limited to English-language publications involving human subjects, with only case reports and case series eligible for inclusion. After a rigorous screening process and data extraction, we conducted a narrative synthesis of the studies. The extracted data are meticulously summarized in Table 1 to facilitate efficient comparison and review. Thirteen papers were reviewed, with eight meeting the inclusion criteria (Figure 2). However, five manuscripts were inaccessible and were therefore excluded from this study.

PTPAs pose significant clinical challenges as evidenced by the case reports summarized in Table 1. Some may be asymptomatic due to spontaneous occlusion or their relatively small size. Kaczynski et al. reported an asymptomatic PTPA measuring 1.3 × 3.6 cm, diagnosed 10 weeks after thromboembolectomy during routine follow-up [14]. In other cases, PTPAs cause local tenderness and present as a painful, swollen, palpable pulsating mass [10,17]. Patients with lower limb edema are often misdiagnosed and treated for deep vein thrombosis [23]. A review of existing BTK cases revealed severe ischemic symptoms—including pallor, paresthesia, and decreased motor function—in four patients [9,11,13,15]. Ischemic foot is one of the most serious complications, potentially leading to limb loss if not promptly addressed. This devastating outcome can result from various pathological processes, such as thrombotic occlusion of the affected artery, embolization from the pseudoaneurysm, or the development of acute lower limb compartment syndrome. Accurate and timely diagnosis is therefore crucial in preventing further complications.

In some cases, symptoms may not appear until weeks or months after the procedure, complicating the diagnosis. Neary et al. documented a case exemplifying this challenge, in which a posterior tibial artery PTPA became symptomatic just 1 week after embolectomy, presenting with severe calf pain and reduced mobility due to the mass effect of the expanding PTPA [11]. The delayed onset of symptoms is often attributed to the gradual dilation of the pseudoaneurysm or the development of complications such as an arteriovenous fistula or arterial rupture. An unusual case involving both an arteriovenous fistula and a pseudoaneurysm in the anterior tibial artery was described by Nenezić et al., with symptoms emerging 1 month post-embolectomy [15]. Additionally, persistent or intermittent bleeding can lead to acute lower limb compartment syndrome or even life-threatening hemorrhage [23]. These cases highlight the need for a high index of suspicion regarding postoperative complications. Duplex ultrasound, CTA, and DSA are essential imaging modalities to prevent these complications from being overlooked because the presence of distal pulses does not rule out arterial perforation, rupture, or arteriovenous fistula formation [10,17].

The treatment plan is influenced by several factors, including the extent and location of the PTPA and the patient’s pre-existing medical conditions. Advances in interventional radiology, particularly embolization techniques, have provided less invasive and effective solutions for managing these vascular injuries. Our review includes seven published reports of endovascular or percutaneous treatment of BTK PTPAs. Coil embolization emerged as the predominant therapeutic approach, used in four cases, all targeting the peroneal artery [10,13,14,17]. Despite its minimally invasive nature, transcatheter embolization carries risks, including distal embolization and inadvertent occlusion of the treated artery or its branches. In addition to transcatheter embolization, covered stents can be deployed to exclude the pseudoaneurysm while preserving arterial flow. The use of covered stents has been documented in the treatment of larger pseudoaneurysms or those associated with arteriovenous fistulas [13].

Percutaneous intervention has proven to be an effective treatment for BTK PTPAs [9,13]. The preference for percutaneous thrombin injection into the pseudoaneurysm sac has increased because of its minimally invasive nature and its ability to preserve critical blood flow. A recent case report involving a posterior tibial artery PTPA demonstrated the efficacy of percutaneous thrombin injection, resulting in complete thrombosis of the pseudoaneurysm while maintaining distal perfusion [11]. Corso et al. published a case in which a patient on anticoagulant therapy was treated with percutaneous thrombin injection using a relatively high volume of thrombin solution without balloon occlusion of the affected artery. This approach successfully induced complete thrombosis of a large pseudoaneurysm with volume of 176.6 cm^3^ while preserving the lumen and flow of the affected BTK artery [9].

One case report documented the successful transcatheter embolization of a peroneal artery PTPA via retrograde puncture of the peroneal artery using platinum microcoils [13]. The insertion of a sheath was crucial in preventing distal coil migration, while the precise placement of the coils ensured the preservation of side branches of the feeding artery [13]. By contrast, open surgical ligation would have posed a higher risk of damaging collateral arteries. The combination of balloon occlusion and coil embolization has proven to be an effective approach for managing wide-necked PTPAs and preventing coil displacement [13,23]. This technique involves temporarily occluding the feeding artery with a balloon, allowing for controlled blood flow and facilitating precise coil placement.

The surgical approach to BTK PTPAs is determined by the extent of arterial damage. Primary closure or vein-patch angioplasty are viable options for repair [24,25], while vein interposition grafting or bypass grafting may also be performed. In a case report by Nenezić et al., a large anterior tibial artery PTPA was successfully replaced with a saphenous vein graft [15]. However, when a concomitant hematoma or injury to surrounding tissue is present, arterial reconstruction can be challenging due to the small caliber of crural arteries. In such cases, ligation of the affected BTK artery should be considered, provided that the remaining crural vessels can adequately perfuse the foot.

In the context of hybrid repair, one case report detailed the combined use of endovascular elimination of the PTPA sac and open surgical anterior leg compartment decompression to manage a complicated anterior tibial artery PTPA. This hybrid approach allowed for immediate control of the PTPA while simultaneously addressing the associated compartment syndrome [12].

None of the cases we reviewed reported complications related to radiological or surgical treatment. All patients were discharged the following day for routine follow-up. Only three case reports included a follow-up period, which otherwise ranged from 1 to 10 months, and no complications such as ischemic events or infections related to PTPA treatment were reported [11,12,15].

This review has shown that the management of PTPAs in BTK arteries presents a significant clinical challenge requiring a multidisciplinary approach. While open surgical repair remains a core treatment option, advances in endovascular techniques offer promising alternatives, particularly for patients who are poor surgical candidates. The choice of treatment modality should be tailored to each patient’s specific needs with consideration of their overall health status, the unique anatomical characteristics of the pseudoaneurysm, and the available medical expertise and resources.

It is important to acknowledge the limitations of this paper, given its nature as a literature review. As such, it is subject to inherent limitations in terms of objectivity, the comprehensiveness of the literature search, and the interpretation of findings.

## 8. Summary

PTPA is a rare complication associated with the use of Fogarty catheters in patients with acute extremity ischemia due to thrombosis or embolism. A comprehensive literature review of case reports published over the past two decades was conducted to provide a concise summary of contemporary management strategies for PTPAs occurring in BTK arteries. Given the limited knowledge in this area, the objective of our review was twofold: first, to highlight current approaches, and second, to emphasize the need for further studies to expand the existing evidence base and improve clinical understanding of the treatment of this infrequent complication.

## Figures and Tables

**Table 1:** Eight analyzed case reports published between January 2000 and May 2024.

Manuscript	Patient	Thromboembolectomy	Time *	PTPA Symptoms	PTPA Diagnosis	PTPA Treatment
Del Grande et al., 2001 [13]	Female; age: 89 years	Left above-the-knee femoropopliteal polytetrafluoroethylene bypass graft	3 days	Painful, enlarging, pulsating hematoma above the ankle	DSA: peroneal artery; size: 2 × 4 cm	Retrograde embolization (direct puncture of the PTPA) with multiple microcoils (d = 2–3 mm)
Neary et al., 2002 [11]	Female; age: 78 years	Right deep and superficial femoral arteries	7 days	Increasingly severe pain in the calf with reduced dorsiflexion	DUS + DSA: posterior tibial artery; size: no data	Thrombin injection (500 IU)
Corso et al., 2003 [9]	Male; age: 65 years	Right branch of axillobifemoral bypass graft and right superficial femoral artery	1 day	Vivid pain and palpable pulsatile mass in the calf, paresthesia of the foot	DUS + DSA: posterior tibial artery; greatest d = 11.5 cm	Thrombin injection (6000 IU)
Sugimoto et al., 2004 [17]	Male; age: 63 years	Right femoropopliteal polytetrafluoroethylene bypass graft	3 days	Hematoma, pain, and pulsating mass in the posterior lower calf	DSA: peroneal artery; size: no data	Occlusion of the feeding artery with microcoils (n = 2, d = 5 mm) proximal to the PTPA
Sadat et al., 2007 [10]	Male; age: 68 years	Right superficial femoral artery	2 weeks	Pain in the lower part of the leg and expansile swelling on the anteromedial surface of the leg, 5 cm above the ankle	DUS + DSA: peroneal artery; size: 5.1 × 2.4 cm	Occlusion of the feeding artery with microcoils (n = 5, d = 4 mm) proximal and distal to the PTPA
Nenezić et al., 2009 [15]	Female; age: 82 years	Right superficial femoral artery	1 month	Painful pulsating mass in the anterior portion of the calf	CTA: anterior tibial artery; size: no data	Great saphenous vein interposition grafting
De Santis et al., 2013 [12]	Male; age: 68 years	Right popliteal artery	2 months	Tender, painful, pulsatile mass in the mid-anterior aspect of the leg	DUS + CTA: anterior tibial artery; size: 3.8 × 6.6 cm	Covered balloon-expandable stent (4 × 19 mm) implantation
Kaczynski et al., 2016 [14]	Male; age: 78 years	Right popliteal artery and tibioperoneal trunk	10 weeks	None	DUS + CTA: peroneal artery; size: 1.3 × 3.6 cm	Occlusion of the feeding artery with microcoils (d = 3–5 mm) proximal and distal to the PTPA

CTA—computed tomography angiography; DSA—digital subtraction angiography; DUS—duplex ultrasound; IU—international unit; PTPA—post-thromboembolectomy pseudoaneurysm. * The time between thromboembolectomy and the diagnosis of PTPA.
